# Intelligent Condition Diagnosis Method Based on Adaptive Statistic Test Filter and Diagnostic Bayesian Network

**DOI:** 10.3390/s16010076

**Published:** 2016-01-08

**Authors:** Ke Li, Qiuju Zhang, Kun Wang, Peng Chen, Huaqing Wang

**Affiliations:** 1 Jiangsu Key Laboratory of Advanced Food Manufacturing Equipment and Technology, Jiangnan University, 1800 Li Hu Avenue, Wuxi 214122, China; like@jiangnan.edu.cn (K.L.); wangkun0808@163.com (K.W.); 2 Graduate School of Bioresources, Mie University/1577 Kurimamachiya-cho, Tsu, Mie 514-8507, Japan; chen@bio.mie-u.ac.jp; 3 School of Mechanical & Electrical Engineering, Beijing University of Chemical Technology, Chao Yang District, Beijing 100029, China; wanghq_buct@hotmail.com

**Keywords:** feature extraction, adaptive statistic test filter, Diagnostic Bayesian Network, evaluation factor, condition diagnosis

## Abstract

A new fault diagnosis method for rotating machinery based on adaptive statistic test filter (ASTF) and Diagnostic Bayesian Network (DBN) is presented in this paper. ASTF is proposed to obtain weak fault features under background noise, ASTF is based on statistic hypothesis testing in the frequency domain to evaluate similarity between reference signal (noise signal) and original signal, and remove the component of high similarity. The optimal level of significance α is obtained using particle swarm optimization (PSO). To evaluate the performance of the ASTF, evaluation factor *I_pq_* is also defined. In addition, a simulation experiment is designed to verify the effectiveness and robustness of ASTF. A sensitive evaluation method using principal component analysis (PCA) is proposed to evaluate the sensitiveness of symptom parameters (SPs) for condition diagnosis. By this way, the good SPs that have high sensitiveness for condition diagnosis can be selected. A three-layer DBN is developed to identify condition of rotation machinery based on the Bayesian Belief Network (BBN) theory. Condition diagnosis experiment for rolling element bearings demonstrates the effectiveness of the proposed method.

## 1. Introduction

In the field of condition monitoring for rotating machinery, the vibration information, such as vibration accelerometer signal, vibration velocity signal, and vibration displacement signal, is often used for detecting faults and distinguishing fault types. Feature extraction of vibration signals is important for condition diagnosis [1,2]. However, feature extraction for condition diagnosis is difficult because the vibration signals measured for condition diagnosis contain strong noise component. Useful information is buried under stronger noise. In such case, the feature of machine condition could not be obtained and even the wrong conclusion will be induced. Thus, it is important that the feature of the signal can be sensitively extracted at the state change of a machine [3]. 

Many studies based on vibration signal processing technology have been carried out with the goal of machinery condition diagnosis [4,5,6,7]. Fourier analysis has been the dominating signal processing tool for condition diagnosis. In [8], Fourier analysis was used to identify the gear faults in planet cage. In [9], Fourier transform has been applied to detect rolling bearing faults. Unfortunately, there are some limitations of the Fourier transform, such as the fact that the signal to be analyzed must be strictly periodic or stationary. However, in practice, machinery operate under unsteady condition, such as varied rotating speed and operating load. In such case, even though the machinery is in the normal state, the spectrum feature and the frequency components of vibration signal are always changing with time. Thus the Fourier transform has no application to analyze non-stationary signal and can not reveal the inherent information of non-stationary signals [10,11]. Time frequency analysis methods, such as Wavelet Transforms (WT), Short Time Fourier Transform (STFT), *etc.*, are effective tools for analyzing the non-stationary signals. These technologies can simultaneously provide the joint distribution information of signals in time domain and frequency domain, and describe the energy density or intensity of the signal at different times and frequencies. In [12], STFT and Hilbert-Huang transform (HHT) analysis were integrated to detect faults of ball bearings for wind turbine. However, the result of the STFT method depends on the choice of the windows size. Moreover, computational cost the STFT is high. WT has got huge success in fault diagnostics of rotating machinery for its ability to focus on localized structures in time frequency domain. WT can decompose the signal into many basis functions and extract signal features through change of the scales and time shifts of the basis function. In [13], WT method was employed to extract fault features of external load changing and the asymmetry of three-phase in induction motor. In [14], the broken-bar fault of induction motor was detected based on discrete WT. However, the feature extraction results of WT rest with the choice of wavelet basis function. Only selecting the appropriate basis function, the features of signal can work well to detect faults. In addition, due to the limited length of the wavelet base function, energy loss is inevitable [15]. Empirical mode decomposition (EMD) technique was proposed by Huang *et al.*, for non-linear and non-stationary signal processing. EMD is a self adaptive signal processing technology that could decompose a non-linear and non-stationary signal into a set of intrinsic mode functions (IMFs). However, undesired frequency components in results and undesired low amplitude IMFs at the low-frequency region remain unsolved in EMD [16,17,18,19]. In addition, there are many noise cancelling methods that have also been applied, such as band pass filter [20], Kalman filter [21], Wiener filter [22], and so on. However, due to their flaws and shortcomings, these methods cannot always be applied to failure feature extraction. For example, band pass filter cannot cancel the wide band noise; when using Wiener filter and Kalman filter to process signal, the signal must follow the normal distribution.

The number of the artificial intelligence techniques, such as artificial neural networks (ANN), ant colony optimization (ACO), Bayesian belief network (BBN) *etc.*, have been widely applied to fault diagnosis of plant machinery. In [23], three architecture NN, single-layer, multilayer perceptron network and counter propagation network, were introduced to detect 10 faults of a heat exchanger. In [2], an improved NN called partially-linearized neural network (PLNN) was presented to distinguishing the three types defect occurred in a rolling bearing. However, NN is not suitable for dealing with ambiguous diagnosis problems, and will never converge if SPs calculated by signals measured in different states have the same value. ACO algorithm imitates the behavior to solve optimization problems. In [24], ACO and DWT were integrated to detect faults of a rolling bearing used in the centrifugal fan system. However, ACO method is easy to trap into local optimum. In many cases, the optimization solution cannot be found. BBN is a powerful tool to represent and reason about complex systems with uncertain, incomplete and conflicting information [25,26]. A BBN enables us to model and reason about uncertainty, ideally suited for diagnosing real world problems where uncertain incomplete data exist. Therefore, it is a suitable solution for troubleshooting complex rotation machinery systems. In the last decades, BBN has been widely applied in condition diagnosis of plant machinery. References [27,28,29] are successful cases of BBN being used to detect fault of complex systems, such as nuclear power systems [27], aircraft engines [28], semiconductor manufacturing systems [29], *etc.*

To extract the fault feature of signals more effectively and discriminate conditions of rotation machinery more correctly, a novel method based on adaptive statistic test filter and Diagnostic Bayesian Network algorithm (DBN) for condition diagnosis of rotating machinery is presented. Structure of this paper is as follows: Section 2 instructs feature extraction method based on ASTF and evaluation factor *I_pq_*. The optimal level of significance α is obtained by using PSO. In Section 3, the ten SPs for condition diagnosis are defined and PCA is employed to obtain high sensitive SPs for condition diagnosis. In Section 4, a three-layer DBN is built to identify condition of rotation machinery based on BBN theory. Section 5 shows a practical example of fault diagnosis for verifying the effectiveness of the proposed method. Summary and conclusions are given in Section 6.

## 2. Feature Extraction by ASTF

**Figure 1:**
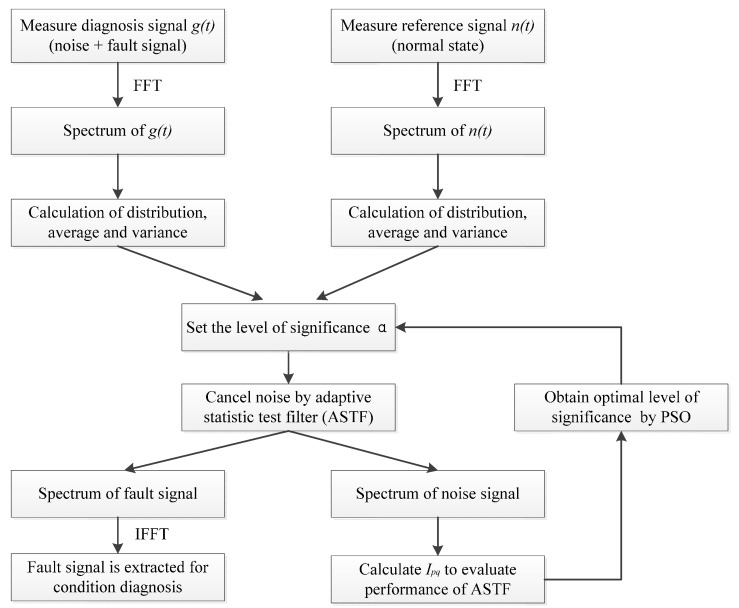
Procedure for applying the ASTF for the condition diagnosis.

In this study, a new weak fault feature extraction method called adaptive statistic test filter (ASTF) is proposed. Principle of ASTF is based on statistic hypothesis testing in the frequency domain to evaluate similarity between reference signal (noise signal) and original signal, and remove the component of high similarity. Otherwise, the optimal level of significance α is obtained using PSO. The procedure for applying STF for the condition diagnosis is proposed, as shown in Figure 1.

The reference signal *n(t)* is measured in a normal state in advance. The original signal *g(t)* is measured in the state to be detected and polluted by noise. $\mu_{n}(f)$ and $\mu_{g}(f)$ indicate the average value of *n(t)* and *g(t)* in the frequency domain, respectively; and $\sigma_{n}^{2}(f)$ and $\sigma_{g}^{2}(f)$ indicate the variance value of *n(t)* and *g(t)* in the frequency domain, respectively. The two null hypotheses are as follows:

(1) $$H_{1}：{\sigma^{2}}_{g}(f)={\sigma^{2}}_{n}(f)$$

(2) $$H_{0}：\mu_{g}(f)=\mu_{n}(f)$$

Firstly, ${\sigma^{2}}_{g}(f)={\sigma^{2}}_{n}(f)$ is verified by *F* test with *m* − 1 degree of freedom, and *F* = *S_n_^2^*/*S_g_^2^*.

(3) $$S_{g}^{2}(f)=\sum_{j=1}^{m}g{\left(f\right)}^{2}/(m-1)-\bar{g}{\left(f\right)}^{2}$$

(4) $$S_{n}^{2}(f)=\sum_{j=1}^{m}n{\left(f\right)}^{2}/(m-1)-\bar{n}{\left(f\right)}^{2}$$

If ${\sigma^{2}}_{g}(f)={\sigma^{2}}_{n}(f)$ , $\mu_{g}(f)=\mu_{n}(f)$ is verified by *t* test with 2*m* − 2 degree of freedom, and $t=\left\{\bar{g}(f)-\bar{n}(f)\right\}/S\sqrt{2/m}$ .

here,

(5) $$S^{2}=\left\{(m-1)S_{g}^{2}+(m-1)S_{n}^{2}\right\}/\left(2m-2\right)$$

If ${\sigma^{2}}_{g}(f)\neq {\sigma^{2}}_{n}(f)$ , $t=\left\{\bar{g}(f)-\bar{n}(f)\right\}/\sqrt{{S_{g}}^{2}\left(f\right)/m\;+\;{S_{n}}^{2}\left(f\right)/m\;}$ .

(6) $$m^{*}={\left(\;{S_{g}}^{2}/m\;+\;{S_{n}}^{2}/m\right)}^{2}/\;\left[\;\left\{\;{S_{g}}^{4}/m^{2}\left(m-1\right)\right\}\;+\;\left\{\;{S_{n}}^{4}/m^{2}\left(m-1\right)\right\}\;\right]$$

If both null hypotheses would prove to be received, the spectrum component of the original signal *g(t)* at the frequency *f* is similar to that of the reference signal *n(t)*. The component at the frequency *f* does not contain fault information and will be removed. If alternative hypothesis is denied, it means that the spectrum component of the original signal *g(t)* at the frequency *f* is not similar to that of the reference signal *n(t)*. The component at the frequency *f* contains fault information.

After STF, the original signal *g(t)* is decomposed into estimated fault signal g^*^(t) and estimated noise signal n^*^(t). In order to appraise the performance of STF, evaluation factor *I_pq_* is defined. *q_i_* and q_i_^*^ are the number that *n(t)* and n^*^(t) cross over some level *i* of the vertical coordinate of the power spectrum *F_n_*^2^(*f_k_*) with a positive slope in unit time and can be calculated as follows:

(7) $$q_{i}=\frac{\sigma_{v}}{2\pi \sigma_{x}}e^{-{n_{i}}^{2}/2{\sigma_{x}}^{2}}$$

(8) $$p_{i}=\frac{\sqrt{2\pi }}{\sigma_{v}}q_{i}$$

(9) $${\sigma_{x}}^{2}=\int_{0}^{\infty }{F_{n}}^{2}(f_{k})\;df_{k}$$

(10) $${\sigma_{v}}^{2}=\int_{0}^{\infty }{\left(2\pi \;f_{k}\right)}^{2}{F_{n}}^{2}(f_{k})\;df_{k}$$

where $i=1\sim K,\;n_{i}=\min\{n(t)\}\sim \max\{n(t)\}$ .

*I_pq_* is defined as follows:

(11) $$I_{pq}=\sum_{i=1}^{K}\;\left|\;\log\left(\frac{q_{i}}{q_{i}*}\right)\;\right|/K+\sum_{i=1}^{K}\;\left|\;\log\left(\frac{p_{i}}{p_{i}*}\right)\;\right|/K$$

It is obvious that the smaller the value of the *I_pq_*, the more similar *n(t)* and n^*^(t) will be, and therefore, the better the STF will be. Thus, *I_pq_* is able to express the similarity degree between *n(t)* and n^*^(t); that is to say, *I_pq_* can be used to evaluate the performance of STF.

To obtain optimal level of significance α, an adaptive PSO algorithm is proposed in this paper. PSO algorithm is based on groups, and solves an unconstrained D-dimensional optimization problem by minimization of the objective or the fitness function [30,31,32]. In this study, the fitness function is the evaluation factor *I_pq_* (Equation (11)). In PSO algorithm, each particle keeps track of its own position denoted by $P(i).location=[X_{i1},X_{i2}\cdots X_{iD}]$ and velocity denoted by $P(i).velocity=[V_{i1},V_{i2}\cdots V_{iR}]$ in the problem space, according to its own and neighboring particle experience [22,23,24]. The best previous position of particle is marked by the lowest fitness value and indicated by $P(i).best=[P_{i1},P_{i2}\cdots P_{iR}]$ . The best position among all particles experienced discovered by the swarm, so far, is defined as $g(i).best=[g_{i1},g_{i2}\cdots g_{iR}]$ . Then, the new positions and velocities of the particles are updated by the following equations:

(12) $$\begin{array}{l} P(i).velocity(t+1)=\omega \;P(i).velocity(t)+\eta_{1}r_{1}[P(i).best(t)-P(i).location(t)]\\ +\eta_{2}r_{2}[g(i)best(t)-P(i).location(t)] \end{array}$$

(13) P(i).location(t+1)=P(i).location(t)+P(i).velocity(t+1)

where *r*_1_ and *r*_2_ indicate random numbers between 0~1. *η_1_* is the cognitive parameter (acceleration coefficient). *η_2_* is the social parameter (acceleration coefficient). The inertia weight *ω* controls the previous velocity of particle, and *ω* adaptively adjust as follows:

(14) $$\omega =\{\begin{array}{c} \begin{array}{cc} k_{1}+0.5q & R>0.05 \end{array}\\ \begin{array}{cc} k_{2}+0.5q & R\leq 0.05 \end{array} \end{array}$$

where *q* is a random number with a uniform probability between 0~1; *k_1_* and *k_2_* are parameters, and *k_1_* > *k_2_* , the choice of *k_1_* and *k_2_* is determined experimentally, here *k_1_* = 0.5 and *k_2_* = 0.2. R indicates change rate, which defined as Equation (15); if *R* is greater than 0.05, PSO is in the exploration stage, a large *ω* is beneficial to the algorithm’s convergence; if *R* is less than 0.05, PSO is in the development stage, a small *ω* is beneficial to searching optimum point.

(15) $$R=\frac{\left|I_{pq}(t+5)-I_{pq}(t)\right|}{\left|I_{pq}(t)\right|}$$

where *I_pq_(t)* is minimization evaluation factor value of the *t*-th iteration. *I_pq_*(*t + 5*) is minimization evaluation factor value of the (*t + 5*)-th iteration. 

In order to test and verify capability of ASTF, a simulation experiment is designed. Ten set signals that consist of the impulsive signal with the period of 0.015 s and random white Gaussian noise are produced using Matlab software to simulate a bearing fault. These noisy signals are processed by ASTF and a high pass filter with 5000 Hz cut off frequency, respectively. The performances of denoising are estimated based on SNR. Mathematical expression of the impulsive signal is shown in Equation (16).

(16) $$x(t)=x_{0}e^{-\xi \omega_{n}t}\sin\omega_{n}\sqrt{1-\xi^{2}t}$$

where *ξ* indicates coefficient of damping and *ξ* = 0.2; *ω_n_* expresses natural frequency and *ω_n_* = 3 kHz; and *x_0_* denotes displacement constant and *x_0_* = 2. 

**Figure 2:**
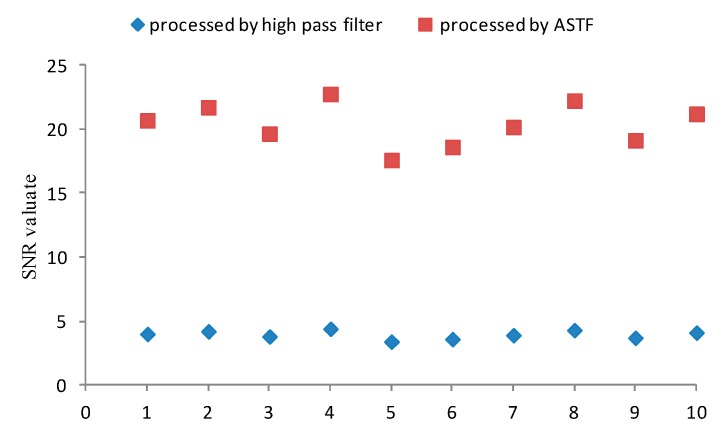
Signal-to-Noise Ratio (SNR) of denoised signals processed by each method.

Figure 2 shows the SNR of denoised signals processed by ASTF and high pass filter. As shown in Figure 2, all of the SNR values of denoised signals after ASTF are much greater than high pass filter. Then, ASTF method is effective and has high robustness for signal denoising.

## 3. Symptom Parameters for Fault Diagnosis and Sensitivity Evaluation

### 3.1. Symptom Parameters for Fault Diagnosis

The number of SPs reflect plant machinery condition have been defined in the pattern recognition field [33]. In this study, ten SPs in the time domain are considered.

(17) $$P_{1}=\frac{\sigma }{\bar{x}}$$

(18) $$P_{2}=\frac{\sum_{i=1}^{N}x_{i}^{2}}{\sigma^{2}}$$

(19) $$P_{3}=\frac{\left|\sum_{i=1}^{N}{\left(x_{i}-\bar{x}\right)}^{3}\right|}{N\sigma^{3}}$$

(20) $$P_{4}=\frac{\sum_{i=1}^{N}{\left(x_{i}-\bar{x}\right)}^{4}}{N\sigma^{4}}$$

where, *x_i_* is digital data of vibration signal. $\bar{x}$ is the mean value of *x_i_*, $\bar{x}=\frac{\sum_{i=1}^{N}x_{i}}{N}$ . σ is standard deviation of *x_i_*, $\sigma =\sqrt{\frac{\sum_{i=1}^{N}{\left(x_{i}-\bar{x}\right)}^{2}}{N-1}}$ .

(21) $$P_{5}=\frac{\bar{x_{p}}}{\bar{x}}$$

(22) $$P_{6}=\frac{\bar{x_{p}}}{\sigma }$$

(23) $$P_{7}=\frac{\left|\sum_{i=1}^{N_{p}}{\left(x_{pi}-\bar{x_{p}}\right)}^{3}\right|}{N_{p}{\sigma_{p}}^{3}}$$

(24) $$P_{8}=\frac{\left|\sum_{i=1}^{N_{p}}{\left(x_{pi}-\bar{x_{p}}\right)}^{4}\right|}{N_{p}{\sigma_{p}}^{4}}$$

where, *x_pi_* is the peak value of *x_i_*. ${\bar{x}}_{p}$ and $\sigma_{p}$ are the mean value and standard deviation of *x_pi_*, respectively.

(25) $$P_{9}=\frac{\left|\sum_{i=1}^{N_{v}}{\left(x_{vi}-\bar{x_{v}}\right)}^{3}\right|}{N_{v}{\sigma_{v}}^{3}}$$

(26) $$P_{10}=\frac{\left|\sum_{i=1}^{N_{v}}{\left(x_{vi}-\bar{x_{v}}\right)}^{4}\right|}{N_{v}{\sigma_{v}}^{4}}$$

where, *x_vi_* is the valley value of *x_i_*. ${\bar{x}}_{v}$ and $\sigma_{v}$ are the mean value and standard deviation of *x_vi_*, respectively.

### 3.2. High Sensitivity Symptom Parameters Obtained by PCA

PCA is a statistical analytical tool used to explore, sort and group data. PCA takes a large number of correlated variables and transform these data into a smaller number of uncorrelated variables known as principal components. The first few principal components contain most of the information and the discriminatory features [34]. 

Define a data matrix with size *m* × *n*, where *m* is the number of identifying states and *n* is the number of SPs, whose covariance matrix has eigenvalue λ_i_ and eigenvector *a_i_* (*a* is loading of the principal component and can express the importance of the SPs for each principal component) and *I* = 1 − *n* with λ_1_ ≥ λ_2_ ≥…≥ λ*_n_*. Principal components *Z_i_* and the cumulative contribution rate of the principal components *η_i_* can be calculated as follows:

(27) $$\left\{\begin{array}{c} Z_{1}\\ \vdots \\ Z_{n} \end{array}\right\}=\left[\begin{array}{ccc} a_{11} & \cdots & a_{1n}\\ \vdots & \ddots & \vdots \\ a_{m1} & \cdots & a_{mn} \end{array}\right]=AP$$

(28) $$\eta_{i}=\sum_{j=1}^{i}\lambda_{j}/\sum_{k=1}^{n}\lambda_{k}$$

where *P_i_* indicates a symptom parameter, *I* = 1 − *n*.

## 4. Bayesian Belief Network

BBN is a probability network based on graphical network model for describing causal uncertainties between variables. It is built for uncertainty modeling and reasoning, and has a great advantage in diagnosing fault caused by uncertainty and correlation of the complex systems.

### 4.1. Bayesian Inference

Supposing *A* is a random event and *B* is the event that is root causes generating *A*, conditional probabilities *P*(*A|B*) between *A* and *B* can be calculated as follows:

(29) $$P(A|\;B)=\frac{P(AB)}{P(B)}=\frac{P(A)P(B|\;A)}{P(B)}$$

where *P*(*AB*) is the joint probability, *P*(*AB*) = *P*(*B*)∙*P*(*A|B*)=*P*(*A*)∙*P*(*B|A*).

Supposing *B_i_* (*i* = 1, 2, ..., *n*) are mutually exclusive and complete set of root causes generating *A*, the marginal probability of *A* is

(30) $$P(A)=\sum_{i=1}^{n}P(B_{i})P(A|\;B_{i})$$

The conditional and marginal probabilities of *A* and *B_i_* is

(31) $$P(B_{i}|\;A)=\frac{P(AB_{i})}{P(A)}=\frac{P(B_{i})P(A|\;B_{i})}{\sum_{i=1}^{n}P(B_{i})P(A|\;B_{i})}$$

In Equation (31), *P(B_i_)* and *P(A*/*B_i_)* express prior probabilities and prior conditional probabilities, respectively. *P(B_i_*/*A)* indicates posterior probability. When using the Bayesian inference for fault diagnosis, *B_i_* represents an equipment condition and *A* represents a SP. The prior probability of the SP*B_i_*(*P*(*B_i_*)) and the conditional probability of equipment condition *A* given *B_i_P(A*/*B_i_)* can be obtained from expert experience or statistical data. Then, the posterior probability *P(B_i_*/*A)* can be obtained by Equation (31). If this posterior probability is high, the condition *B_i_* can be confirmed at the given *A*, and the equipment condition is judged *B_i_*.

### 4.2. Topology of Bayesian Belief Network

**Figure 3:**
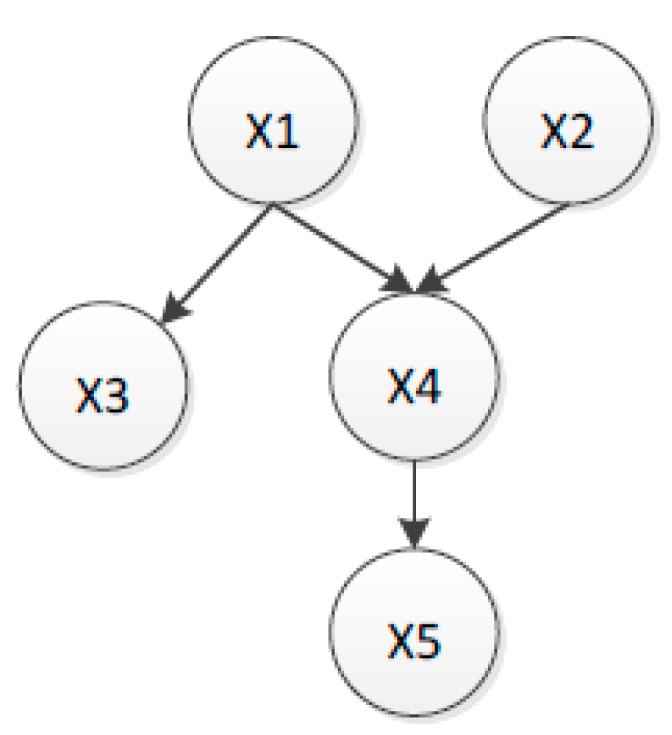
General model of Bayesian Belief Network.

A BBN consists of a number of nodes, directed links, and probability tables. For a diagnostic BBN model, nodes represent variables that can be SP, equipment condition or observations. Directed links indicate casual relationships between the variables. In this paper, the purpose of building a diagnostic BBN is to reason the most likely mechanical condition based on the values of SP, given one or more SP values to calculate posterior probabilities of the cause. The calculus of posterior probability involves calculating the joint probability for the model (probabilities of all combined states for all nodes within the model). The network contains five nodes, *X*1*, X*2*, X*3*, X*4*,* and *X*5, with a structure of three layers (see in Figure 3). In terms of the definition of the three types of conditional independence, *X*1 is independent of *X*2; *X*1 is parent of *X*3 and *X*4. Given *X*1, *X*3 and *X*4 are conditionally independent of each other, *X*5 is independent of *X*1*, X*2*,* and *X*3. The following derivation indicates how to calculate the posterior conditional probability *P(X*4 = true|*X*5 = true*)*.

(32) $$P(X_{4}=\text{true}|\;X_{5}=\text{true})=\frac{P(X_{4}=\text{true}|\;X_{5}=\text{true})}{P(X_{5}=\text{true})}=\frac{\sum_{x_{1}x_{2}x_{3}}P(X_{1}，X_{2}，X_{3}，X_{4}=\text{true},X_{5}=\text{true})}{\sum_{x_{1}x_{2}x_{3}x_{4}}P(X_{1}，X_{2}，X_{3}，X_{4},X_{5}=\text{true})}$$

where *P(X*1*, X*2*, X*3*,X*4 = true|*X*5 = true*)* and *P(X*1*, X*2*, X*3*,X*4 ,*X*5 = true*)* involve calculating the joint probability of the model. The joint probability of this model *P(X*1*, X*2*, X*3*, X*4*, X*5*)* can be calculated as follows:

(33) $$\begin{array}{l} P(X_{1}，X_{2}，X_{3}，X_{4},X_{5})=\prod_{i=1}^{5}P(X_{i}|\;X_{1},\cdots ,X_{i-1})\\ =P(X_{1})P(X_{2}|\;X_{1})P(X_{3}|\;X_{1}X_{2})P(X_{4}|\;X_{1}X_{2}X_{3})P(X_{5}|\;X_{1}X_{2}X_{3}X_{4}) \end{array}$$

Applying the independence assumption, the joint probability distribution can be simplified as follows:

(34) $$\begin{array}{l} P(X_{1}，X_{2}，X_{3}，X_{4},X_{5})=\prod_{i=1}^{5}P(X_{i}|P_{ai})\\ =P(X_{1})P(X_{2})P(X_{3}|\;X_{1})P(X_{4}|\;X_{1}X_{2})P(X_{5}|\;X_{4}) \end{array}$$

### 4.3. Framework of Diagnostic Bayesian Network

**Figure 4:**
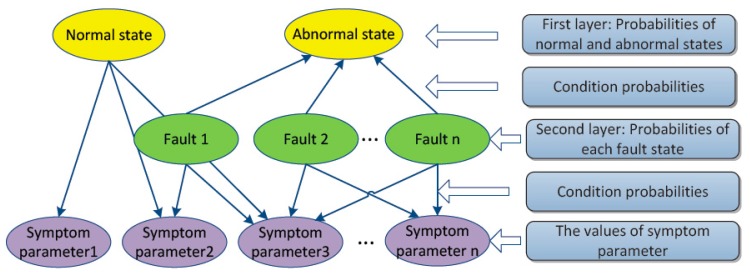
Structure of Diagnostic Bayesian Network.

In this study, Diagnostic Bayesian Network (DBN) is constructed for intelligent condition diagnosis. As shown in Figure 4, the proposed DBN consists of three layers. The first layer is normal and abnormal states. The second layer is fault states, and the last layer is SPs calculated from the signals processed by ASTF. 

In the proposed DBN, prior probabilities of root nodes are needed and conditional probabilities are also needed to represent direct probabilistic dependences among nodes in the three layers. Here, all machine states are regarded as parent nodes, and the prior probabilities of state *i* (*S_i_*) can be obtained as follows:

(35) $$P(S_{i})=\frac{N_{S_{i}}}{N}$$

where *N_Si_* represents the sample size of state *i*, and *N* indicates the total number of samples.

The conditional probabilities of each node are obtained as follows

(36) P(SP=xi| Si)=NSixiNSiNSixi≠0P(SP=xi| Si)=1NNSi+NxiNifNSixi=0

where *SP* represents the values of symptom parameters, and $N_{S_{i}}^{x_{i}}$ indicates the sample size of state *i*, when *SP* = *x_i_*. 

## 5. Diagnosis and Application

### 5.1. Condition Diagnosis by Proposed Method

**Figure 5:**
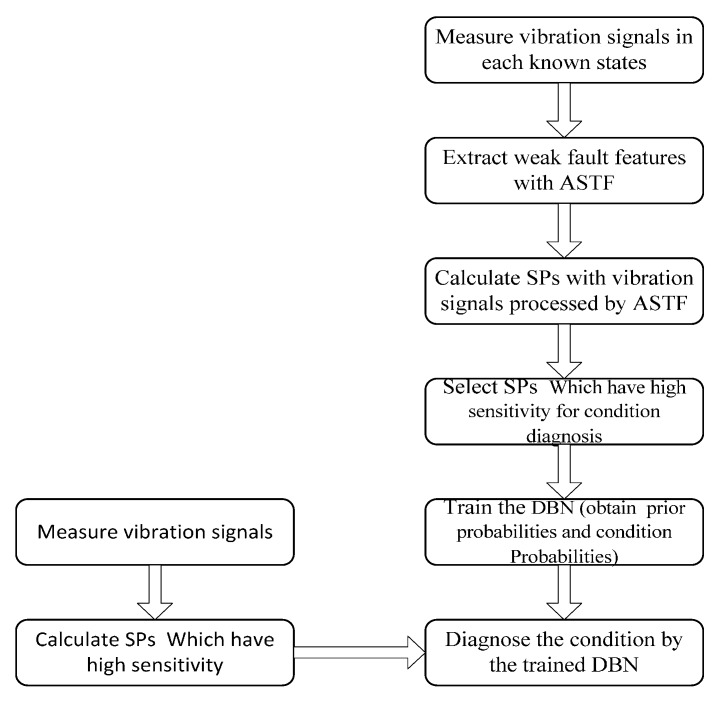
Flowchart for the condition diagnostic procedure.

In this section, an experimental setup is designed to evaluate the effectiveness of the method proposed in this paper. The flowchart of the condition diagnostic procedure is shown in Figure 5.

**Figure 6:**
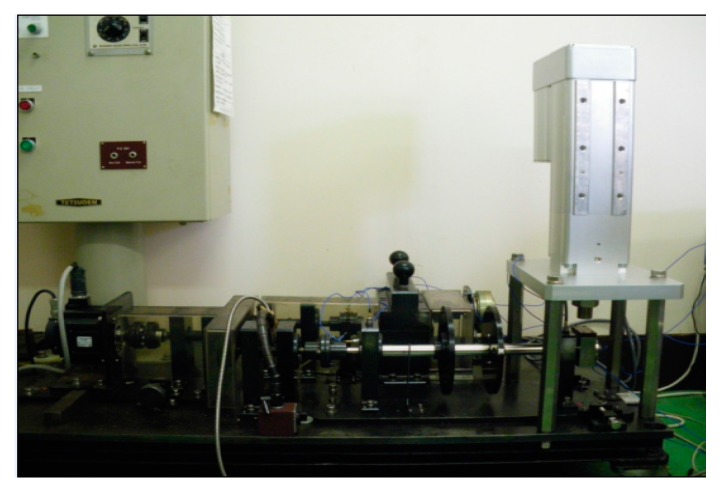
Experimental system for bearing fault diagnosis.

**Figure 7:**
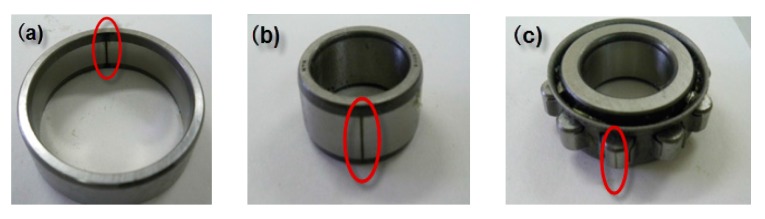
Bearing defects: (**a**) outer-race defect; (**b**) inner-race defect; and (**c**) roller defect.

Figure 6 shows the experimental bench for condition diagnosis test, which includes a servo motor, rotor system and loading equipment. The NSK 205 ball bearing is used for bearing condition diagnosis. As shown in Figure 7, three types fault: the outer defect, the inner defect, and the roller element defect were artificially made by using electro discharge machining with fault width was 0.3 mm, and fault depth was 0.025 mm. 

**Figure 8:**
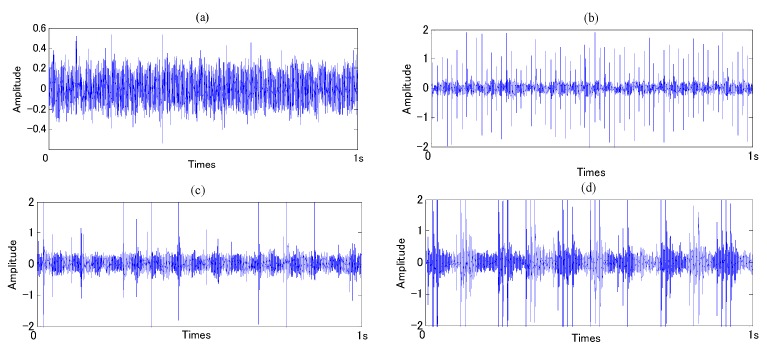
Original vibration signal: (**a**) normal state; (**b**) outer-race defect; (**c**) inner-race defect; and (**d**) roller defect.

**Figure 9:**
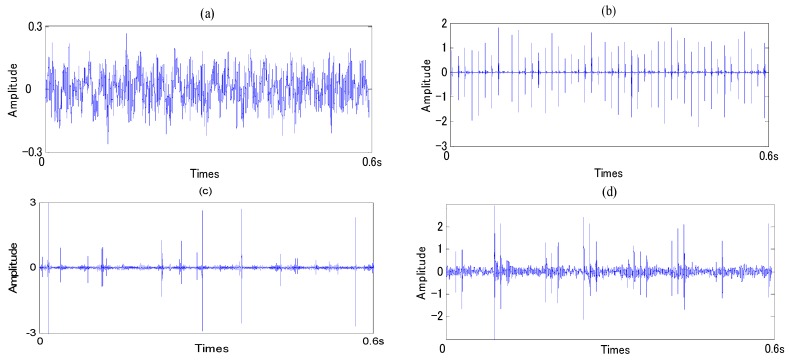
Vibration signal after ASTF: (**a**) normal state; (**b**) outer-race defect; (**c**) inner-race defect; and (**d**) roller defect.

In the present work, the original vibration signals in each state were measured by the accelerometer (PCB MA352A60, PCB Piezotronics Inc., New York, NY, USA) with 50,000 Hz sampling frequency. The accelerometer was fixed on vertical direction of the bearing. While the vibration signals were being obtained, the speed of servo motor was 800 rpm, and a 150 kg load was also transported on the rotating shaft by the loading equipment (RCS2-RA13R, IAI Co. Ltd., Shizuoka, Japan). All the data were recorded and transformed by a collection system includes a sensor signal conditioner (PCB ICP Model 480C02, PCB Piezotronics Inc., New York, NY, USA) and a signal recorder (Scope Coder DL750, YOKOGAWA Co. Ltd. Tokyo, Japan). Obtained data was divided to two sets, one set includes 80 samples and was used to train diagnosis system; the other set includes 20 samples and was used for condition identification test. Figure 8 shows the original vibration signal in each state, and Figure 9 shows the vibration signal after ASTF.

In this study, the SPs that contain the most information and have high sensitivity for each state are selected by PCA. As an example, parts of the selection results are shown in Table 1 and Table 2; P_1_, P_2_, P_6_, P_8_ and P_9_ have high sensitivity for distinguishing normal state and abnormal state. Because the weight coefficients for P_1_, P_2_, P_6_, P_8_ and P_9_, the first principal component, are larger than those of the other, the contribution rate of the first principal component is larger than 0.86, which contains enough information and discriminatory features to identify the normal state and abnormal state. Similarly, the SPs for other states can also be selected.

**Figure 10:**
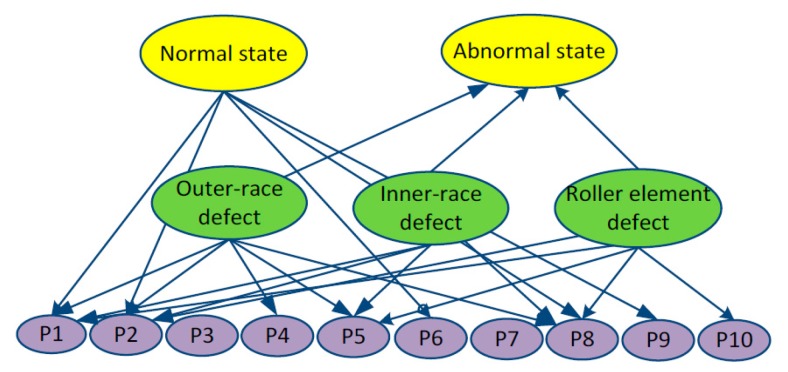
DBN for distinguishing conditions of a rolling bearing.

In this paper, the DBN for distinguishing conditions of a rolling bearing was built as shown in Figure 10. The proposed DBN consists of three layers. The first layer is normal and abnormal states. The second layer is main failures such as outer-race defect, inner-race defect, and roller element defect, which often occurred in a rolling bearing. The last layer is SPs shown in Table 2. The prior probabilities and the conditional probabilities were obtained by Equations (35) and (36). All of the SPs were divided into five levels, 1, 2, 3, 4 and 5, which indicate very small, small, middle, big and very big levels, respectively. As an example, parts of the training sample data are shown in Table 3. 

**Table 1:** First principal component of SPs.

	Weight Coefficients for Each Symptom Parameter									Contribution Rate	
	P_1_	P_2_	P_3_	P_4_	P_5_	P_6_	P_7_	P_8_	P_9_	P_10_	*η*_1_
N:O	0.99	0.91	−0.62	−0.75	−0.33	0.85	−0.69	0.88	0.79	0.21	0.88
N:I	0.93	0.87	−0.53	−0.32	−0.65	0.78	0.11	0.92	0.81	−0.42	0.86
N:R	0.99	1.0	−0.9	−0.36	−0.75	0.9	−0.22	0.86	0.91	−0.36	0.89
O:I	0.87	1.0	−0.56	0.99	0.88	−0.66	−0.71	0.98	-0.56	0.38	0.90
O:R	0.99	0.99	−0.37	0.93	0.95	−0.58	−0.62	0.98	-0.79	0.86	0.85
I:R	0.97	0.86	−0.78	0.36	0.91	−0.55	−0.11	0.87	-0.91	0.95	0.86

**Table 2:** Selection result of the SPs for distinguishing each state.

State	Selection result
Normal	P_1_, P_2_, P_6_, P_8_, P_9_
Outer-race defect	P_1_, P_2_, P_4_,P_5_, P_8_,
Inner-race defect	P_1_, P_2_, P_5_, P_8_,
Roller-element defect	P_1_, P_2_, P_5_, P_8_, P_10_,

**Table 3:** Training sample data.

State	Non-dimensional Symptom Parameters									
	P_1_	P_2_	P_3_	P_4_	P_5_	P_6_	P_7_	P_8_	P_9_	P_10_
Normal	1	1	1	1	1	1	1	1	1	1
	1	1	1	1	1	2	1	1	1	2
	1	1	1	1	1	2	1	2	1	1
	1	1	1	1	1	1	1	1	1	2
	...	...	...	...	...	...	...	...	...	...
Outer-race defect	2	3	1	4	4	3	1	3	2	3
	3	3	1	4	4	3	1	3	2	4
	2	2	1	3	4	3	1	3	2	5
	3	3	1	4	4	3	1	3	2	3
	...	...	...	...	...	...	...	...	...	...
Inner-race defect	3	1	2	1	1	2	1	3	4	4
	4	1	2	2	1	5	5	4	5	4
	3	1	1	2	2	4	3	4	4	4
	3	1	1	1	1	3	3	3	3	2
	...	...	...	...	...	...	...	...	...	...
Roller element defect	5	5	1	3	5	5	3	4	4	5
	4	4	2	4	5	5	3	5	5	5
	4	4	5	3	5	5	3	4	3	5
	4	4	2	3	5	5	3	4	4	5
	...	...	...	...	...	...	...	...	...	...

To verify the diagnostic capability of the diagnosis methods proposed in this paper, we used the data measured in each state had not been used to train the DBN system. They can correctly and quickly diagnose those faults with the possibility grades of the corresponding states. In the test of normal state, the successful diagnosis ratio is 100%. In the test of each faults, the successful diagnosis ratio of outer-race defect, inner-race defect and roller element defect states are 100%, 94% and 86%, respectively. Some diagnosis results are shown in Table 4, Table 5, Table 6 and Table 7.

**Table 4:** Diagnosis results of normal state.

Non-Dimensional Symptom Parameters					State		Judge
P_1_	P_2_	P_6_	P_8_	P_9_	Normal	Abnormal	
1	1	1	1	1	0.96	0.04	Normal
1	1	1	2	1	0.89	0.11	Normal
1	1	2	1	1	0.91	0.09	Normal
...	...	...	...	...	...	...	...

**Table 5:** Diagnosis results of outer-race defect state.

Non-Dimensional Symptom Parameters					State		Judge
P_1_	P_2_	P_4_	P_5_	P_8_	Outer-Race Defect	Other Faults	
3	3	4	4	3	0.99	0.01	Outer-race defect
2	2	4	4	3	0.89	0.11	Outer-race defect
2	3	5	4	3	0.86	0.14	Outer-race defect
...	...	...	...	...	...	...	...

**Table 6:** Diagnosis results of inner-race defect state.

Non-dimensional Symptom Parameters				State		Judge
P_1_	P_2_	P_5_	P_8_	Inner-Race Defect	Other Faults	
4	1	2	3	0.85	0.15	Inner-race defect
3	2	1	4	0.79	0.21	Inner-race defect
3	1	2	4	0.88	0.12	Inner-race defect
...	...	...	...	...	...	...

**Table 7:** Diagnosis results of roller element defect state.

Non-dimensional Symptom Parameters					State		Judge
P_1_	P_2_	P_5_	P_8_	P_10_	Roller-Element Defect	Other Faults	
4	4	5	5	5	0.75	0.25	Roller-element defect
3	3	4	5	5	0.68	0.32	Roller-element defect
3	4	5	4	5	0.66	0.34	Roller-element defect
...	...	...	...	...	...	...	...

### 5.2. Condition Diagnosis by NN

**Figure 11:**
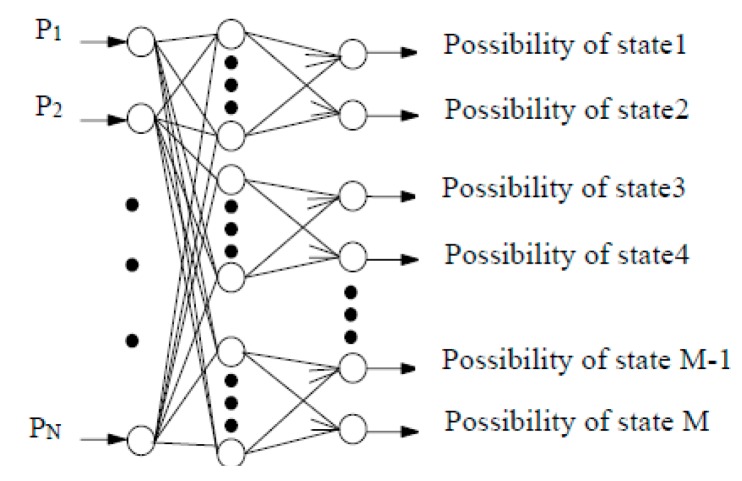
The back propagation NN for condition diagnosis.

In this study, a back propagation NN shown in Figure 11 is also constructed for condition diagnosis of the roller bearing. The NN consists of three layers, the SPs calculated by vibration signals are entered into input layer, hidden layer includes 80 units, output layer is the possibility grades of each condition of roller bearing. Table 8 shows the parts of diagnosis results of NN. N, O, I and R indicate the normal, outer race defect, inner race defect and roller element defect states, respectively. The symbol × expresses the case that NN is incapable of identifying the fault type. As shown in Table 8, the normal and outer race defect states of roller bearing were correctly identified by NN. However, NN is incapable of identifying inner race defect and roller element defect states of roller bearing. The main reasons are that the vibration signals measured for condition diagnosis contain strong noise, there exist ambiguous relationships between the SPs and the fault types, and NN cannot deal with incomplete and conflicting information.

**Table 8:** Diagnosis results of NN.

P_1_	P_2_	P_3_	P_4_	P_5_	P_6_	P_7_	P_8_	P_9_	P_10_	N	O	I	R	Judge
0.02	0.06	0.13	6.13	2.24	1.0	1.98	4.72	2.01	11.9	0.868	0.001	0.135	0.012	N
0.02	0.07	0.11	5.87	2.58	1.03	1.75	5.73	1.15	10.1	0.895	0.001	0.098	0.169	N
0.03	0.16	0.15	103	4.85	2.01	2.33	66.3	3.05	105	0.063	0.805	0.177	0.055	O
0.03	0.17	0.19	106	4.62	2.15	2.68	72.5	3.66	99.7	0.087	0.796	0.206	0.036	O
0.02	0.11	0.25	33.4	2.91	3.06	10.8	80.7	8.49	109	0.056	0.071	0.532	0.405	×
0.04	0.09	0.32	48.9	3.65	1.95	9.67	85.6	8.98	94.9	0.095	0.041	0.501	0.386	×
0.05	0.32	0.47	93.9	5.29	3.57	8.87	115	10.8	124	0.011	0.095	0.406	0.513	×
0.06	0.26	0.68	106	5.47	3.26	9.13	123	10.2	139	0.032	0.086	0.366	0.572	×
...	...	...	...	...	...	...	...	...	...	...	...	...	...	...

## 6. Conclusions

In order to detect the condition of rotating machinery at an early stage, a novel fault diagnosis method based on ASTF and DBN was presented. The main conclusions of this paper are summarized as follows:

1. The method of ASTF for extracting weak fault features under background noise was presented. The optimal level of significance α was obtained using PSO. To evaluate the performance of ASTF, evaluation factor *I_pq_* was also defined. In addition, a simulation experiment was designed to verify the effectiveness and robustness of ASTF.
2. PCA based on statistical analysis theory was also presented to evaluate the sensitivities of SPs calculated via vibration signals measured in each state for condition identification.
3. A three-layer DBN was developed to identify condition of rotation machinery based on the BBN theory. It is effective and efficient in condition diagnosis based on uncertain, incomplete and conflicting information.
4. Study examples of diagnosis for a bearing were shown to demonstrate the effectiveness of the methods proposed in this paper. The verification results show that the bearing faults that often occur in roller bearings, such as the Outer race, the Inner race and the roller element defects, have been effectively identified by the proposed method in this paper. However, these bearing faults are difficult to detect using NN technology, because the vibration signals measured for condition diagnosis contain strong noise, there exist ambiguous relationships between the SPs and the fault types, and NN cannot deal with incomplete and conflicting information.

In summary, this paper verifies the capability of condition diagnosis method based on ASTF and DBN. In addition, soft sensor technique establish inference model of symptom parameters based on state-space model, and solves inference model of symptom parameters by parameter estimation method, such as Kalman filter and Bayesian filter. The condition diagnosis system includes two parts: feature extraction and condition identification. Soft sensor technique can be used of feature extraction and fusion, and condition identification can adopt artificial intelligence techniques, such as DBN, NN, *etc.* In the future, we will consider using soft sensor technique to extract features of signals.
